# Comparing national infectious disease surveillance systems: China and the Netherlands

**DOI:** 10.1186/s12889-017-4319-3

**Published:** 2017-05-08

**Authors:** Willemijn L. Vlieg, Ewout B. Fanoy, Liselotte van Asten, Xiaobo Liu, Jun Yang, Eva Pilot, Paul Bijkerk, Wim van der Hoek, Thomas Krafft, Marianne A. van der Sande, Qi-Yong Liu

**Affiliations:** 1 Department of Health, Ethics & Society, CAPHRI School of Public Health and Primary Care, Faculty of Health, Medicine and Life Sciences, Maastricht University, Maastricht, The Netherlands; 2 Centre for Infectious Diseases, Epidemiology and Surveillance, National Institute for Public Health and the Environment, Bilthoven, The Netherlands; 3 Public Health Service, region Utrecht, Zeist, The Netherlands; 4 State Key Laboratory of Infectious Disease Prevention and Control, Collaborative Innovation Centre for Diagnosis and Treatment of Infectious Diseases, National Institute for Communicable Disease Control and Prevention, Chinese Centre for Disease Control and Prevention, Beijing, 102206 China; 5 Julius Centre for Primary Care and Public Health, University Medical Centre, Utrecht, The Netherlands

**Keywords:** Risk assessment and early warning, Infectious disease surveillance systems, Surveillance of unexplained pneumonia, Arthropod borne virus disease surveillance, China, The Netherlands

## Abstract

**Background:**

Risk assessment and early warning (RAEW) are essential components of any infectious disease surveillance system. In light of the International Health Regulations (IHR)(2005), this study compares the organisation of RAEW in China and the Netherlands. The respective approaches towards surveillance of arboviral disease and unexplained pneumonia were analysed to gain a better understanding of the RAEW mode of operation. This study may be used to explore options for further strengthening of global collaboration and timely detection and surveillance of infectious disease outbreaks.

**Methods:**

A qualitative study design was used, combining data retrieved from the literature and from semi-structured interviews with Chinese (5 national-level and 6 provincial-level) and Dutch (5 national-level) experts.

**Results:**

The results show that some differences exist such as in the use of automated electronic components of the early warning system in China (‘CIDARS’), compared to a more limited automated component in the Netherlands (‘barometer’). Moreover, RAEW units in the Netherlands focus exclusively on infectious diseases, while China has a broader ‘all hazard’ approach (including for example chemical incidents). In the Netherlands, veterinary specialists take part at the RAEW meetings, to enable a structured exchange/assessment of zoonotic signals.

**Conclusion:**

Despite these differences, the main conclusion is that for the two infections studied, the early warning system in China and the Netherlands are remarkably similar considering their large differences in infectious disease history, population size and geographical setting. Our main recommendations are continued emphasis on international corporation that requires insight into national infectious disease surveillance systems, the usage of a One Health approach in infectious disease surveillance, and further exploration/strengthening of a combined syndromic and laboratory surveillance system.

## Background

In view of the complex and global spread of infectious diseases, the timely detection of outbreaks requires mechanisms to increase situational awareness and to initiate outbreak management. Outbreaks such as Zika (2015), Ebola (2014) and severe acute respiratory syndrome (SARS) (2003) have shown the necessity of effective infectious disease surveillance systems for early detection, to allow proper assessment, a fast response and collaboration at regional, national and global levels [1]. Infectious disease surveillance systems are an important source for early warning and depend on data from laboratory tests, clinical diagnoses and syndromic sources, among others [2, 3].

To strengthen infectious disease surveillance in the aftermath of the 2003 SARS outbreak and to control diseases at their source, the adjusted International Health Regulations (IHR) (2005) aimed for a syndrome-inclusive approach that encouraged surveillance of infectious diseases of both known and unknown origin. The IHR aims to enhance early warning systems for World Health Organization (WHO) Member States that contribute to global communication. To enable timely communication between WHO Member States and the WHO itself, National IHR Focal Points are set up. These Focal Point are responsible for notifying WHO on events that may constitute a Public Health Emergency of International Concern (PHEIC) [4].

Early warning systems are also in place on a supranational and (sub-)national level. To our knowledge, there are no standard early warning protocols among countries, but many countries have established units to screen surveillance sources, in order to be able to assess and control infectious disease outbreaks. In this study, we refer to these units as Risk Assessment and Early Warning (RAEW) units, although we are aware this is not a standard term or universally applied concept. RAEW units usually organize recurring, fixed meetings among infectious disease experts to discuss observations from (inter)national surveillance systems, to inform public health professionals and the public and to initiate outbreak management. Information exchange mechanisms are often in place with supranational agencies such as the European Centre for Disease Prevention and Control (ECDC) to strengthen defences against infectious diseases.

Although Chinese early warning systems have improved greatly since the SARS outbreak (2003) and the Netherlands expanded their early warning systems after their experience with a large Q fever outbreak (2007–2009) [5, 6], further lessons can be learned. This study describes how RAEW units in both China and the Netherlands approach/utilise infectious disease surveillance. We analysed notable differences and similarities to understand the systems in place. In-depth studies focusing on surveillance of arthropod borne viruses (arboviruses) disease and unexplained pneumonia were included as examples. The first is an example of an common illness and the second is an example of a uncommon illness in both countries. In light of the IHR, this comparison provides insights into the functioning of infectious disease surveillance and early warning systems in different settings, and can be used for exploring options for further strengthening of international collaboration, timely detection and surveillance of infectious diseases.

## Methods

A qualitative study design was used through a literature search and semi-structured interviews to acquire in-depth knowledge on early warning systems in a global perspective, with a specific focus on arbovirus and unexplained pneumonia surveillance systems in China and the Netherlands. Scientific databases (Pubmed, BioMed Central, Informa Healthcare and Google Scholar) were searched using keywords related to early warning. In the Netherlands, 5 experts from RIVM were interviewed. In China, 6 experts from the Chinese Centre for Disease Control and Prevention (China CDC) and 5 experts from the Beijing provincial CDC were interviewed. The interview questions were structured according to the guidelines for evaluating public health surveillance systems [7] and included a general description of infectious disease surveillance systems used by RAEW units, the operation of the surveillance system, surveillance sources and involved parties.

## Results

**Fig. 1:**
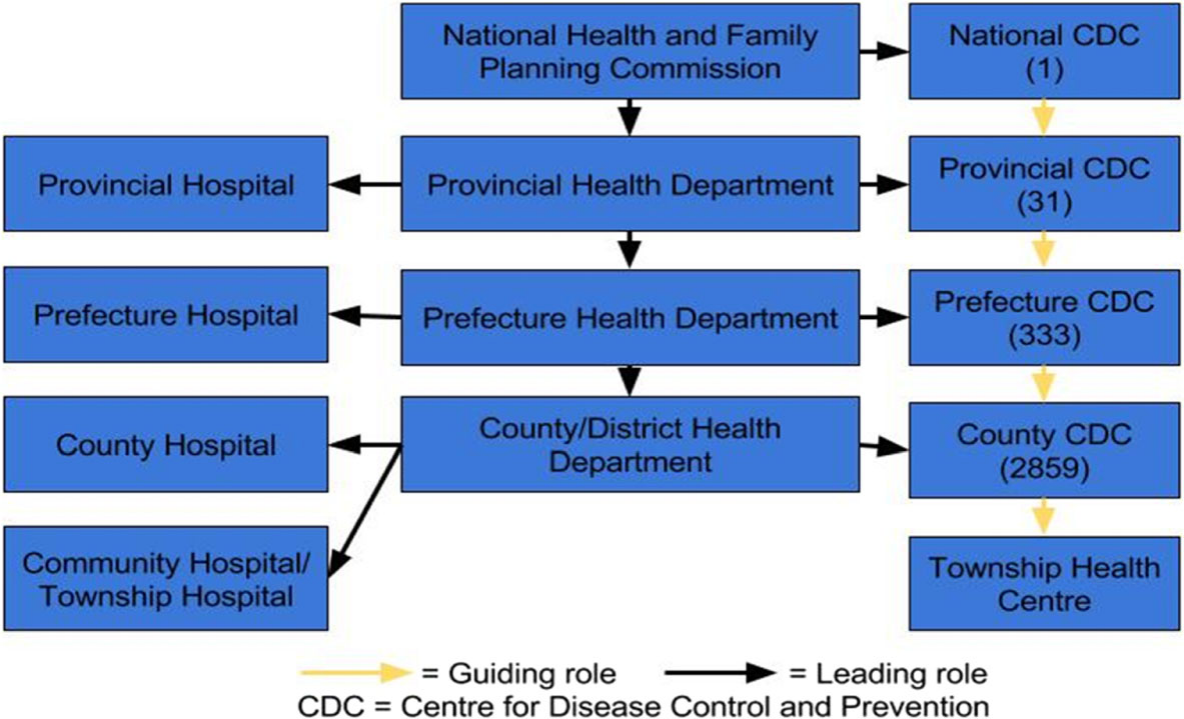
Organization of Chinese public health organizations involved in infectious disease control

China (population 1.3 billion) and the Netherlands (population 17 million) both operate multi-layered infectious disease surveillance systems. In China, infectious disease surveillance data is analysed by the China CDC at different levels: national, provincial, prefecture and county (Fig. 1) [8, 9]. The overarching institute is the National Health and Family Planning Commission (NHFPC), which is in charge of prevention and treatment of infectious diseases and nationwide supervision. Sub-national health departments are in charge of similar tasks within their own administrative areas [10].

**Fig. 2:**
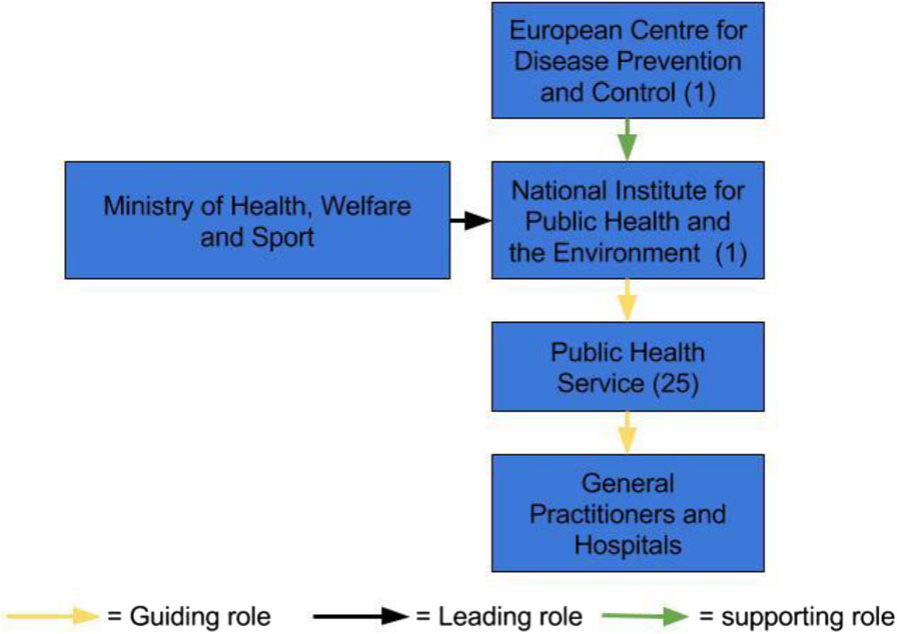
Organization of Dutch public health organizations involved in infectious disease control

In the Netherlands, infectious disease surveillance data is analysed nationally by the RIVM and regionally by public health services (PHS) (Fig. 2). A PHS is the leading body for disease outbreak management if the outbreak is restricted to local administrative regions and national coordination is not required. When national coordination is necessary, the Ministry of Health, Welfare and Sport (VWS) and RIVM are the key actors. The ECDC has a supporting role towards the Netherlands in order to strengthen Europe’s defences against infectious diseases.

### Notifiable infectious disease reporting systems

As China and the Netherlands are Member States of WHO, events that may constitute a PHEIC and certain diseases (e.g. polio and SARS) require international reporting under the IHR [11]. In China, the National IHR Focal Point is situated at the NHFPC. In the Netherlands, it is situated at RIVM. With the exception of diseases that have to be monitored according to the IHR, the list of notifiable diseases is determined nationally.

#### Categorization of notifiable diseases

In China, 39 infectious diseases are notifiable by law and categorized as A, B or C diseases [12]. Category A diseases should be reported within 2 h of diagnosis, others within 24 h [10]. China CDC guidelines also require reporting of several additional diseases (e.g. unexplained pneumonia and Zika virus infections). In the Netherlands, 43 diseases and 3 conditions are notifiable and are categorized as A, B1, B2 or C diseases [13]. Category A diseases should be reported immediately, others should be reported within 24 h [14]. Most notifiable infections are similar between the two countries but are in different notification categories (Table 1). Certain differences can be explained by specific epidemiological and geographical situations, such as for arboviruses. For example, Japanese encephalitis virus infection and dengue virus infection are notifiable in China, but not in the Netherlands. Further, seasonal influenza, some sexually transmitted diseases and HIV are notifiable in China, but not in the Netherlands. Legal measures that can be taken vary by category. Category A diseases allow for the same legislative measures in both countries, including patient isolation and hospitalization.

**Table 1:**
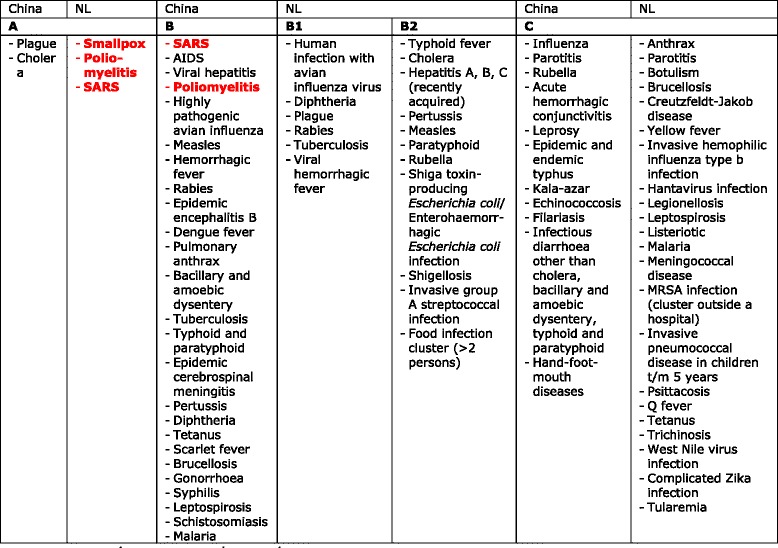
Notifiable diseases in China and the Netherlands [10, 11, 13]

In red and bold: diseases that require reporting under the IHR (2005)

#### Logistics of reporting notifiable diseases

In China, if a notifiable infectious disease is clinically diagnosed and/or laboratory confirmed according to the unified national diagnostic criteria issued by the NHFPC, cases must be reported to the national China CDC, which collects and analyses the acquired data. The health care provider enters the case information using a standard form into the Notifiable Infectious Diseases Reporting Information System (NIDRIS), a web-based system that enables all healthcare institutions to report cases of notifiable infectious diseases. Approximately 5 million infectious disease cases are reported annually (≈ 385 cases per 100,000 citizens per year) [8]. Each China CDC level can analyse its own data in NIDRIS and data from subordinate levels within its own administrative boundaries.

In the Netherlands, if a notifiable infectious disease is suspected and/or laboratory tests confirms it, the case must be reported both by the attending physician and the laboratory to the regional PHS. The case information is collected and entered by the PHS into Osiris, a web-based database that transmits the data to RIVM for further analyses. In 2014, 13,863 notifiable disease cases were reported via Osiris to RIVM (≈ 815 cases per 100,000 citizen per year) [15].

### Risk assessment and early warning

Timeliness is an important factor for an effective response [16]. It requires surveillance, assessment and communication mechanisms to increase situational awareness and to initiate outbreak management. To reach this, RAEW units in China and the Netherlands are using several (inter)national infectious disease sources based on multiple data collection methods.

**Fig. 3:**
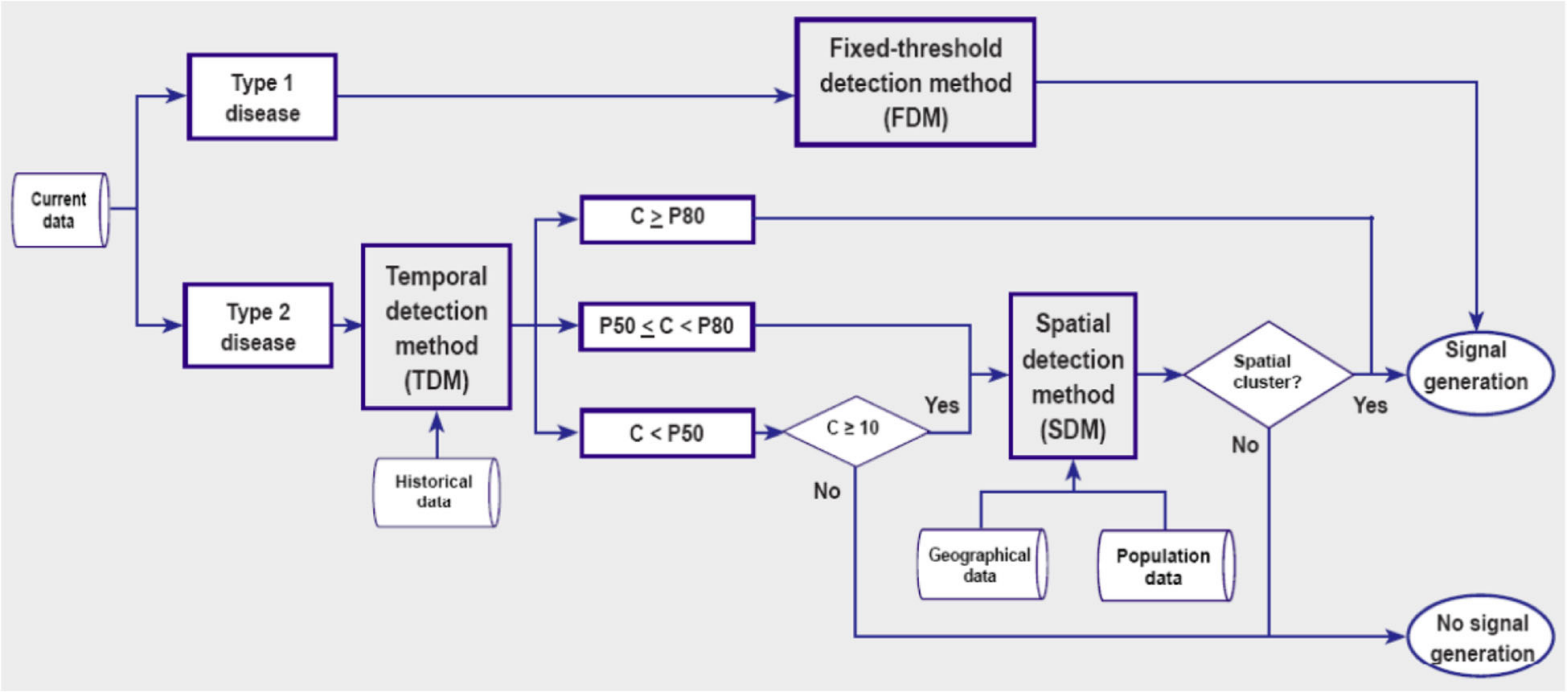
The aberration detection and signal technology flow chart of the infectious disease automated alert and response system, as visualized by Yang et al. [8]

To facilitate early warning at different China CDC levels, the China Infectious Disease Automated-alert and Response System (CIDARS) has been in place since 2008 [8]. This system consists of four components: aberration detection, signal generation, signal dissemination and signal response information feedback. It is based on the surveillance data of 33 of the 39 notifiable diseases in NIDRIS (chronic diseases are excluded). The diseases are divided in a type 1 or type 2 disease: type 1 diseases have a higher severity but lower incidence and type 2 diseases include more common infectious diseases. For type 1 diseases, a fixed-threshold detection method with real-time monitoring is used. In case of type 2 diseases, a temporal and/or spatial detection method with daily monitoring is in place (Fig. 3). When a signal is generated, it will be reported to county-level China CDCs in the affected regions by Short Message Service (SMS). After receiving the signal by SMS, the county-level specialists conduct signal verification and field investigation to confirm an outbreak. The conclusions from field investigations are entered into CIDARS [8, 17]. Although CIDARS is seen as sensitive and effective, challenges remain regarding the proportion of false positive signals and the sheer amount of SMS signals that are distributed.

**Fig. 4:**
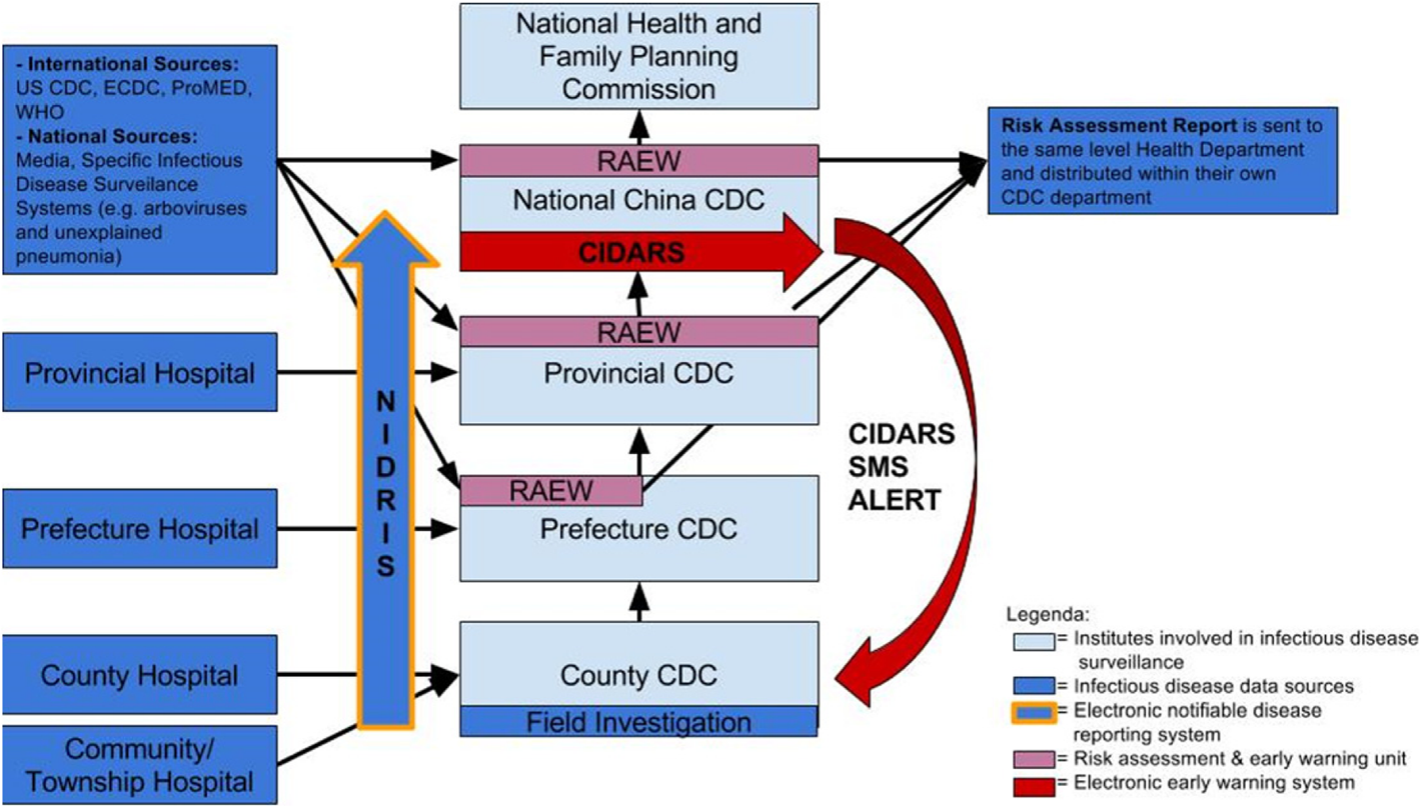
Flow chart risk assessment and early warning in China

Although the Chinese early warning system is based on CIDARS, China CDC also organizes regular RAEW meetings among infectious disease experts (Fig. 4). Important signals are discussed and outbreak management is discussed. The signals are derived from various (inter)national sources such as NIDRIS, CIDARS, specific infectious disease surveillance systems, United States CDC and ECDC. There is a daily risk assessment meeting that discusses new signals, a monthly risk assessment conference discussing major signals and a disease/risk factor conference (only organized when needed) discussing specific threats that may require further in-depth understanding. Different types of events can be discussed at these meetings: infectious disease signals, natural disasters and environmental and occupational threats. These events are mostly human health related, since there is limited collaboration with the veterinary sector. Similar meetings can also be conducted at the provincial and prefecture level. After each RAEW meeting, an alert report with relevant signals is produced and sent to the NHFPC, to the same level health departments and within China CDC.

**Fig. 5:**
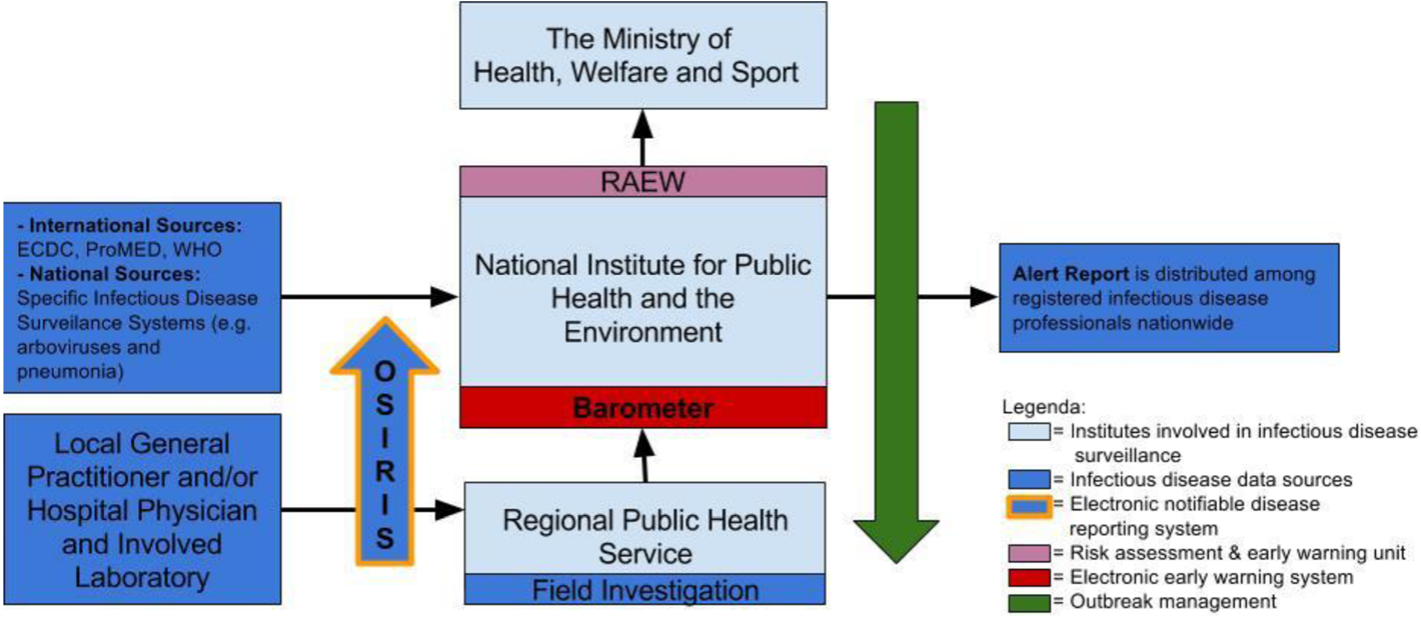
Flow chart risk assessment and early warning in the Netherlands

In the Netherlands, the RIVM organizes regular RAEW meetings to discuss important signals and advise on or initiate an outbreak response (Fig. 5). The signals are derived from (inter)national sources, such as Osiris, ECDC daily round table reports, WHO, the PHS and ProMED [18]. Moreover, Dutch experts use the ‘barometer’ algorithm that compares the number of infectious disease notifications in the past 4 weeks with the expected value [19, 20]. In contrast to CIDARS, the ‘barometer’ does not send a SMS when a threshold is exceeded, as this would generate too many false positive signals and increase the workload unnecessarily. Moreover, due to privacy concerns and data protection regulations, the ‘barometer’ does not automatically include geographical data and therefore has no spatial detection method. The RAEW meetings that use the above sources are the weekly Netherlands early warning committee (NEWC) meeting that focuses on infectious disease signals, a monthly zoonosis meeting and a monthly hospital and antimicrobial resistance meeting. If needed, a response meeting is organized as a follow-up, whereby a specific signal can be discussed in-depth. The discussed events are mostly human and animal health related; environmental threats are not part of routine discussions. After each NEWC meeting, an alert report with relevant signals is produced and sent by weekly email to over 2400 professionals involved in infectious disease control (e.g. doctors and medical microbiologists). After each zoonosis and hospital and antimicrobial resistance meeting, a monthly report is sent to the involved physicians. Information exchange with EU partners is facilitated through cooperating with ECDC.

### In-depth studies

To better understand the workflow and to provide a detailed explanation of the surveillance systems focusing on a specific disease, in-depth studies were performed on arbovirus and unexplained pneumonia surveillance in China and the Netherlands.

#### Unexplained pneumonia surveillance

Although lower respiratory infections, including pneumonia, are one of the main causes of death worldwide [21], real-time surveillance systems and situational awareness are generally lacking.

In the year after the SARS outbreak in 2003, NHFPC developed a surveillance system for unexplained pneumonia to facilitate timely detection of airborne pathogens that form a severe threat to public health. Therefore, all Chinese health care facilities are required to report any patient who has a clinical diagnosis of pneumonia with an unknown causative pathogen and whose illness meets the following five criteria (2007 modified definition): (1) fever ≥38 °C; (2) radiologic characteristics consistent with pneumonia; (3) normal or reduced leukocyte count or low lymphocyte count in early clinical stage; (4) no improvement or worsening of the patient’s condition after first-line antibiotic treatment for 3–5 days; and (5) the pneumonia etiology cannot be attributed to an alternative laboratory or clinical diagnosis (clinicians are granted flexibility to determine how to interpret this criterion and specific tests are not specified) [22, 23]. Once the case is registered in NIDRIS, the data are further analysed in CIDARS as a type 1 disease, for which a fixed-threshold method (of 1 case) is applied. A real-time SMS is followed by a field investigation, whereby case samples are tested to rule out avian influenza, SARS and Middle East respiratory syndrome coronavirus (MERS-CoV). Although physicians are required to report unexplained pneumonia cases, considerable under-reporting occurs [22]. The aim of this surveillance system is not to detect each unexplained pneumonia case but to focus on clusters that could indicate an (unknown) emerging infectious disease outbreak.

Unexplained pneumonia is not a notifiable condition in the Netherlands as it is in China. However, according to the Public Health Act (2008), each physician should notify a case or an unusual number of cases with an (unknown) infectious disease that forms a severe threat to public health. An example is the Q fever outbreak (2007); the unusual number of atypical pneumonia cases early in the outbreak were not detected by routine surveillance systems but by astute general practitioners (GPs) [24]. Both Dutch legislation and the Chinese pneumonia surveillance system aim for early notification of (unknown) emerging infectious disease outbreaks. However, in both countries, criteria for notification are not well defined and a considerable degree of under-ascertainment and under-reporting is likely. In the Netherlands, structural syndromic pneumonia surveillance is carried out using data extracted from electronic patient files maintained by sentinel GP practices, representing 7% of the Dutch population. Moreover, sentinel registration of pneumonia cases in nursing homes takes place [25]. A separate virologic laboratory surveillance system provides information on circulating respiratory viruses. Since 2015, a pilot study has been carried out for hospitalized severe acute respiratory infections (SARI) patients. As it includes only two of 133 hospitals in the country at present, the obtained data is not yet reliable to provide early warning of infectious pneumonia outbreaks. Currently, no set threshold exists for unusual occurrence of pneumonia. Expert opinion determines which signals are discussed by the NEWC.

#### Arbovirus disease surveillance

The current Zika outbreak (2015) shows how important an effective arbovirus surveillance system is, as the IHR requires countries to report unusual Zika-related cases [26].

In China, dengue virus infection and Japanese encephalitis virus infection are to be reported (both category B) [12]. Japanese encephalitis is believed to be the only endemic arbovirus in China [27, 28]. Cases of Zika virus infection, chikungunya virus infection and severe fever with thrombocytopenia syndrome virus should also be reported according to guidelines of China CDC but are not listed in the law as notifiable disease. Once a case of these arboviruses is detected and entered into NIDRIS, it is analysed in CIDARS. For example, dengue virus infection is categorized as a type 2 disease for which a temporal detection method is applied. This detection method compares reported cases in the current observation period (in the past 7 days) to the previous 5 years at the county level. When the number of reported cases in the observation period reaches the predefined threshold and when there is a spatial cluster, a SMS will be disseminated and followed by field investigation [8]. The arbovirus surveillance system is mainly based on laboratory tests in China, since syndromic surveillance for arboviruses is carried out only in a few provinces.

In the Netherlands, West Nile virus (WNV) infection, transmitted via endemic Culex mosquitoes, is currently the only notifiable arbovirus infection (category C) [13]. Once a case is confirmed, the physician reports the case to the PHS, where after the PHS enters the information into Osiris. The Kingdom of the Netherlands also includes several overseas islands in the Caribbean: the Caribbean municipalities (Bonaire, Sint Eustatius and Saba) and the sovereign territories (Aruba, Curacao and Sint Maarten). In the Caribbean municipalities, WNV infection, dengue virus infection and chikungunya virus infections are currently notifiable; the latter two are endemic. In the case of a diagnosis, the patient must be reported as category C notifiable disease. Under-reporting occurs as a result of the often mild course of the diseases, rarely performed laboratory tests and workload issues. In the Caribbean municipalities and the Caribbean sovereign territories, syndromic surveillance plays an important role in increasing situational awareness. The number of patients with fever and respiratory symptoms are reported to the officer of the Caribbean surveillance system by the majority of GPs. Weekly trends per island are sent to the Caribbean Public Health Agency and to RIVM. The data used by the NEWC includes an overview of reported cases via Osiris from the Netherlands and the Caribbean municipalities, combined with syndromic data from the GPs in the Caribbean municipalities.

## Discussion

The results of the present study indicate that many similarities exist among infectious disease surveillance for early warning systems in China and the Netherlands. Both countries generally apply similar notifiable disease reporting systems and RAEW units. The infectious disease surveillance institutes have comparable aims and functionalities in China and the Netherlands, which reflects 100% IHR (2005) implementation.

Some differences exist in early warning systems. In China, early warning is mainly based on the automatically generated and disseminated signals of CIDARS by means of fixed thresholds. In the Netherlands, the emphasis is on expert opinion based on epidemiological analysis and additional checking of raw data. The experts at RIVM decide whether or not to contact PHS for further investigation. Multiple factors need to be taken into account to understand why certain systems are in place. At first, the infectious disease outbreak history plays an important role. The Netherlands experienced Q fever outbreaks (2007–2009) [5, 29], leading to a dedicated zoonosis early warning committee. In China, there were more than 5300 SARS cases during the 2003 outbreak [30], which resulted in the development of the automated NIDRIS and CIDARS systems. Moreover, the scope of infectious disease surveillance influenced the development of early warning systems. Since China has the largest population worldwide, CIDARS provides a tool to analyse the large volume of reported data and to rapidly inform the county level without the need for expert opinion at the national level [8]. We might ask whether an automatic SMS signal dissemination would also be beneficial for the Dutch ‘barometer’. Currently the need for such a SMS system to regional PHS’s is not supported, as the existing early warning structure can inform stakeholders in time, without a large number of false positive automatic signals that would drain resources and negatively affect commitment. This is mainly possible because of the limited number of outbreaks and short communication lines between government and medical professionals in the Netherlands. But there is always a risk that an outbreak is being overlooked. As this is a first overview, additional efforts will be needed to develop methods to improve the sensitivity and specificity of the existing alert systems.

The concept of ‘One Health’ emphasized by the Dutch government recognizes that in such a densely populated country, the health of humans is interlinked with the health of animals and the environment and acknowledges the importance of intensive animal husbandry [31]. China could explore if there would be added value in stronger connection with the veterinary sector by including animal infectious disease signals in their RAEW meetings. While RAEW units in the Netherlands focus exclusively on infectious diseases, China however has a broader ‘all hazard’ approach. Inclusion of other health signals, such as chemical incidents, is not yet considered in the Netherlands, since a different structure exists for these public health hazards. However, at the European level, there is increasing emphasis on legislation focusing on a wide range of communicable and non-communicable health threats in the context of preparedness and early warning [32].

Unexplained pneumonia and arbovirus surveillance in China relies more on laboratory confirmation than the Dutch surveillance system, where syndromic surveillance currently plays a larger role. Since China is a large country with multiple governmental levels, the spectrum of socio-economic conditions and facilities for laboratory testing vary widely among different regions [33]. To increase the situational awareness in areas where laboratory testing is limited, the potential of syndromic surveillance could be explored. For the Netherlands (including the Caribbean islands), improving the laboratory capacity for unknown emerging arboviruses could be beneficial since this is a potential risk as demonstrated by the chikungunya and Zika outbreaks (2015) [34, 35]. Different unexplained pneumonia surveillance approaches are used in both countries, but the objectives are similar. Further development of SARI surveillance for hospitalized patients in the Netherlands (for which a pilot study is currently being carried out) may assist timely detection of respiratory outbreaks.

## Conclusions

The infectious disease surveillance systems for the two diseases assessed in China and the Netherlands are remarkably similar in general structure considering the large differences in the two countries’ infectious disease history, population size and geographical setting. Routine procedures and (electronic) communication mechanisms are key components in the system, and help both countries to achieve situational awareness and to control infectious disease outbreaks.

However, the systems differ on some details. The substantial demographic differences and recent history of emerging infectious disease outbreaks in both countries may have influenced the assessment and communication mechanisms in place. The main differences are the usage of thresholds and automatically top-down disseminated (SMS) signals in China for validation purposes, which might be more efficient and perhaps better accepted in a large country. This is in contrast to a more qualitative and exploratory approach in the Netherlands, probably due to its small size and short communication lines. Those surveillance differences must be addressed at the international reporting level, in order to convey national used baselines or syndrome counts in understandable terms. Continued emphasis is therefore needed on international cooperation to curb the global spread of infectious diseases, which requires an insight into the early warning and infectious disease surveillance systems of all countries to improve global assessment and response capabilities.

The additional zoonotic RAEW in the Netherlands, initiated since a large Q-fever epidemic, improved zoonotic surveillance and assessment capacity. Therefore we recommend that infectious disease surveillance systems should consider using a One Health approach, to interlink the environment, human and animal health. While the Chinese ‘all hazard’ approach might improve the surveillance assessment quality due to early involvement of different stakeholders and experts, with shared public health responsibilities.

Combining syndromic surveillance together with the outcomes of laboratory tests increases the probability of timely detection and proper assessment. Further strengthening a combined syndromic and laboratory surveillance system can be further explored by both countries.
